# Galcanezumab in episodic migraine: the phase 3, randomized, double-blind, placebo-controlled PERSIST study

**DOI:** 10.1186/s10194-022-01458-0

**Published:** 2022-07-28

**Authors:** Bo Hu, Gang Li, Xiaohong Li, Shan Wu, Tingmin Yu, Xiang Li, Hongru Zhao, Zhihua Jia, Junpeng Zhuang, Shengyuan Yu

**Affiliations:** 1 Department of Neurology, Union Hospital, Tongji Medical College, Huazhong University of Science and Technology, Wuhan, China; 2 Department of Neurology, Shanghai East Hospital, School of Medicine, Tongji University, Shanghai, China; 3 Department of Neurology, Jinan Central Hospital, Cheeloo College of Medicine, Shandong University, Jinan, China; 4 Department of Neurology, Affiliated Hospital of Guizhou Medical University, Guiyang, Guizhou China; 5 Department of Neurology, The Second Hospital of Jilin University, Changchun, Jilin China; 6 Department of Neurology, Huashan Hospital, Shanghai Medical College, Fudan University, Shanghai, China; 7 Department of Neurology, The First Affiliated Hospital of Soochow University, Suzhou, China; 8 Department of Neurology, Chinese PLA General Hospital, Beijing, China; 9 Eli Lilly and Company, Shanghai, China

**Keywords:** Calcitonin gene-related peptide, Galcanezumab, Humanized monoclonal antibody, Episodic migraine

## Abstract

**Background:**

Galcanezumab, a humanized monoclonal antibody that binds calcitonin gene-related peptide, has demonstrated efficacy and good tolerability in patients with episodic migraine in previous phase 3 trials. We report results from the PERSIST study, which was designed to assess the efficacy and safety of galcanezumab in patients with episodic migraine from China, India, and Russia.

**Methods:**

This phase 3 study was conducted at 40 centers in China (*n =* 26), India (*n =* 10), and Russia (*n =* 4). Eligible adult patients with episodic migraine were randomized in a 1:1 ratio to receive monthly galcanezumab 120 mg (with 240 mg loading dose) or placebo during a double-blind, 3-month treatment period. The primary endpoint was the overall mean change from baseline in monthly migraine headache days (MHDs). Key secondary endpoints were the mean proportion of patients with ≥ 50%, ≥ 75%, and 100% reductions from baseline in MHDs and mean change in the Migraine-Specific Quality of Life Questionnaire (MSQ) Role Function-Restrictive domain score.

**Results:**

In total, 520 patients were randomized and received at least one dose of galcanezumab (*N =* 261) or placebo (*N =* 259). The least squares (LS) mean reduction from baseline in monthly MHDs over 3 months was significantly greater with galcanezumab compared with placebo (-3.81 days vs. -1.99 days; *p <* 0.0001). Significantly greater mean proportions of patients with galcanezumab versus placebo had ≥ 50%, ≥ 75%, and 100% reductions from baseline in MHDs (all *p <* 0.0001). The overall mean improvement from baseline in MSQ Role Function-Restrictive score over 3 months was significantly greater with galcanezumab versus placebo (*p <* 0.0001). There were no clinically meaningful differences between the galcanezumab and placebo group on any safety parameters except for a higher incidence of injection site pruritus (5.0% vs. 0.0%), injection site reaction (3.8% vs. 0.4%), and injection site discomfort (2.3% vs. 0.0%). TEAEs related to injection sites were mild in severity, except in 1 patient who had a moderate injection site reaction. Six serious adverse events were reported by 6 patients (2 galcanezumab, 4 placebo).

**Conclusions:**

Galcanezumab 120 mg once monthly was effective and well tolerated in patients with episodic migraine from China, India, and Russia.

**Trial registration:**

ClinicalTrials.gov Identifier NCT03963232 (PERSIST), registered May 24, 2019.

## Introduction

Migraine is a common neurological disorder, and was ranked as the second leading cause of years lived with disability worldwide in 2016 [1]. In China, the 2017 age-standardized rates (per 100,000) of years lived with disability from migraine and migraine prevalence were 331 and 9211, respectively [2]. Although it is estimated that 34% to 39% of people who experience migraines should be considered for preventive treatment [3, 4], only 2.7% to 15.0% of Chinese migraine patients receive preventive medicine [5, 6]. Current preventive migraine medications include beta-blockers, anticonvulsants, antihypertensives, and antidepressants [7, 8]. However, these treatments were designed for use in other indications and are associated with low rates of adherence and persistence in patients with migraine, largely due to a lack of efficacy and poor tolerability [9, 10]. Therefore, new preventive treatments with improved efficacy and tolerability are required.

Calcitonin gene-related peptide (CGRP) is widely expressed throughout the central and peripheral nervous system and is implicated in the pathophysiology of migraine [11]. Serum concentrations of CGRP are elevated during migraine attacks, [12, 13] and infusion of CGRP to individuals with a history of migraine can trigger a migraine attack [14]. Targeting CGRP therefore represents a rational approach in migraine prevention and treatment. Monoclonal antibodies against CGRP or its receptor have demonstrated efficacy in preventing migraine attacks and may have a more favorable benefit-risk profile compared with established preventive treatments for episodic migraine [15, 16].

Galcanezumab is a humanized monoclonal antibody that binds CGRP and prevents its biological activity without blocking the CGRP receptor. Data from pivotal phase 3 trials have demonstrated the efficacy and tolerability of galcanezumab compared with placebo in patients with episodic migraine (EVOLVE 1 and 2), [17, 18] chronic migraine, [19] and previous migraine preventive medication failures of two to four categories (CONQUER) [20]. In patients with episodic migraine, galcanezumab 120 mg and 240 mg significantly reduced the mean number of monthly migraine headache days (MHDs) compared with placebo by 4.7 and 4.6 days versus 2.8 days, respectively (both *p <* 0.001) in the EVOLVE 1 study [17], and by 4.3 and 4.2 days versus 2.3 days, respectively (both *p <* 0.001) in the EVOLVE 2 study [18]. In both studies, galcanezumab 120 mg and 240 mg demonstrated favorable tolerability, with low rates of discontinuation due to adverse events [17, 18]. However, the patient populations in these two studies were predominantly Caucasian and data on galcanezumab are lacking in Asian patients with episodic migraine.

The PERSIST study was designed to assess the efficacy and safety of galcanezumab in patients with episodic migraine from China, India, and Russia. This article reports the results from the 3-month, double-blind treatment period of this study.

## Methods

### Study design and treatment

**Fig. 1:**
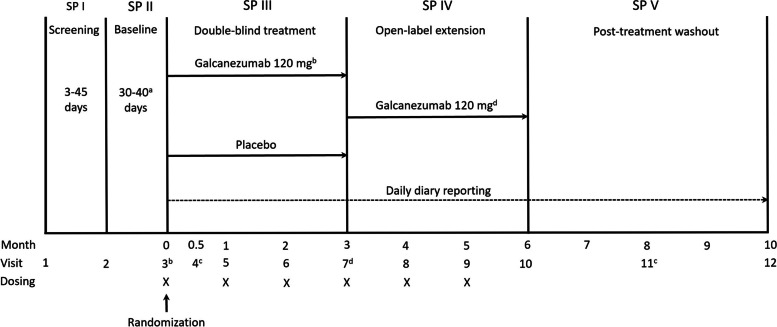
Study design. ^a^Eligibility period determined between a minimum of 30 days and a maximum of 40 days. ^b^Patients randomized to galcanezumab 120 mg received a loading dose of 240 mg at the first injection only (Visit 3). ^c^Telephone visits. dAt Visit 7, patients randomized to placebo who entered the open-label extension received galcanezumab at a loading dose of 240 mg, while patients randomized to galcanezumab 120 mg continued treatment at the 120 mg dose. SP, study period; X indicates when dosing occurred

This was a phase 3 study conducted at 40 centers in China (*n =* 26), India (*n =* 10), and Russia (*n =* 4). The study comprised five periods: initial screening and washout of all excluded medications or migraine preventive treatments taken prior to study entry (3–45 days); a prospective baseline period to determine patient eligibility based on daily entries into an electronic patient-reported outcomes (ePRO) system (30–40 days); a 3-month, randomized, double-blind, placebo-controlled treatment period; a 3-month open-label extension; and a 4-month post-treatment phase to observe the washout of the study drug (Fig. 1). Here, we report the results of the double-blind period; the database lock was August 2021. Results from the open-label and post-treatment periods will be reported separately.

During the double-blind treatment period, patients were randomized 1:1 to receive monthly subcutaneous injections of galcanezumab 120 mg (with a loading dose of 240 mg) or placebo during an office visit. Patients could continue to take the following acute migraine medications: acetaminophen (paracetamol), non-steroidal anti-inflammatory drugs, triptans, ergotamine and derivatives, isometheptene mucate, dichloralphenazone and acetaminophen combination (Midrin), or combinations thereof. Assignment to treatment was via a computer-generated random sequence using an interactive web-response system. Randomization was stratified by country and baseline migraine frequency (< 8 MHDs vs. ≥ 8 MHDs per month). To preserve blinding, patients received two injections at the beginning of the randomized period (two galcanezumab 120 mg injections or two placebo injections). Galcanezumab and placebo (excipients only) were supplied as visually indistinguishable 1-ml, single-dose, prefilled, disposable manual syringes with study-specific labels.

### Patients

Eligible patients were aged 18 to 65 years at the time of screening, with a diagnosis of episodic migraine as defined by the third edition of the International Classification of Headache Disorders (ICHD-3; 1.1 or 1.2) [21], a history of migraines for ≥ 1 year prior to Visit 1, and migraine onset before 50 years of age. Patients were required to have a history of 4–14 MHDs and ≥ 2 migraine attacks per month on average within the 3 months prior to Visit 1, and a frequency of 4–14 MHDs and ≥ 2 migraine attacks during the prospective baseline period. To avoid bias, patients were not told the number of MHDs on which study qualification was based. Patients had to be at least 80% compliant with ePRO daily diary entries during the prospective baseline period.

Patients were excluded if they had failed to respond to three or more classes of migraine preventive treatments, were currently receiving treatment for migraine prevention, or were taking or expected to take therapeutic antibodies during the study. Other key exclusion criteria were prior exposure to galcanezumab or another CGRP antibody, known hypersensitivity to multiple drugs, serious or unstable psychiatric conditions, history of stroke, risk for acute cardiovascular events based on medical history or electrocardiogram findings, and body mass index ≥ 40 kg/m^2^.

### Assessments and endpoints

Patients used the ePRO system each day to record headache information, migraine-associated symptoms, and the use of headache medication. The Migraine-Specific Quality of Life Questionnaire (MSQ) version 2.1, which consists of 14 items across three domains of patient functioning scored from 0 to 100, with scores of < 40 indicating extreme impairment and scores of 85–100 indicating no or minimal impairment [22], was administered at randomization (baseline) and month 1, 2 and 3. The Patient Global Impression of Severity (PGI-S) scale [23], which measures severity of illness with scores ranging from 1 (normal, not at all ill) to 7 (extremely ill), and the Migraine Disability Assessment (MIDAS) score [24], which consists of five items measuring headache-related disability ranging from little or no disability (0–5) to severe disability (> 20), were administered at baseline and month 3. Safety measures included treatment-emergent adverse events (TEAEs), serious adverse events (SAEs), death, discontinuations due to adverse events, vital signs, and weight. Immunogenicity measures included antidrug antibodies (ADA), neutralizing ADAs, and treatment-emergent ADAs.

The primary endpoint was the overall mean change from baseline in the number of monthly MHDs during the 3-month, double-blind period. Key secondary endpoints were the proportion of patients with ≥ 50%, ≥ 75%, and 100% reduction from baseline in monthly MHDs during the 3-month, double-blind period and overall mean change from baseline through months 1 to 3 in the MSQ Role Function-Restrictive domain score. Other secondary endpoints included mean changes from baseline in the number of monthly MHDs treated with acute migraine medication, MSQ total score, Role Function-Preventive and Emotional Function domain scores, PGI-S score, MIDAS total score, safety, and immunogenicity.

### Statistical analysis

In total, approximately 486 patients were planned for randomization. It was estimated that 243 patients per treatment group would provide approximately 90% power to detect an effect size of 0.33 between the galcanezumab and placebo groups, at an overall 2-sided 0.05 significance level, assuming a discontinuation rate of 20% during the double-blind period.

Efficacy and safety analyses included all patients who underwent randomization and received at least one dose of study drug. The primary endpoint and continuous secondary efficacy endpoints were analyzed using a restricted maximum likelihood-based MMRM technique including the fixed categorical effects of treatment, country, month, and treatment-by-month interaction, as well as the continuous fixed covariates of baseline value and baseline-by-month interaction. Repeated binary efficacy endpoints were estimated using a categorical, pseudo-likelihood-based repeated measures analysis implemented using a generalized linear mixed model procedure including the fixed categorical effects of treatment, month, and treatment-by-month interaction, as well as the continuous, fixed covariate of baseline value. If a significant improvement in the number of monthly MHDs was observed at month 1 and maintained through to month 3, it was planned to analyze the number of weekly MHDs using an ordinal repeated measures model. The incidence of TEAEs and immunogenicity parameters were summarized by treatment group.

To provide strong control of the study-wise type I error rate, the primary and key secondary endpoints were tested using a gated approach at a 2-sided alpha level of 0.05. If the null hypothesis was rejected for the primary endpoint, the key secondary endpoints were to be sequentially tested in the following order: ≥ 50% reduction from baseline in monthly MHDs, MSQ Role Function-Restrictive domain score,  ≥ 75% and 100% reduction from baseline in monthly MHDs. The statistical evaluation was performed using SAS version 9.4 or higher.

## Results

### Patients

**Fig. 2:**
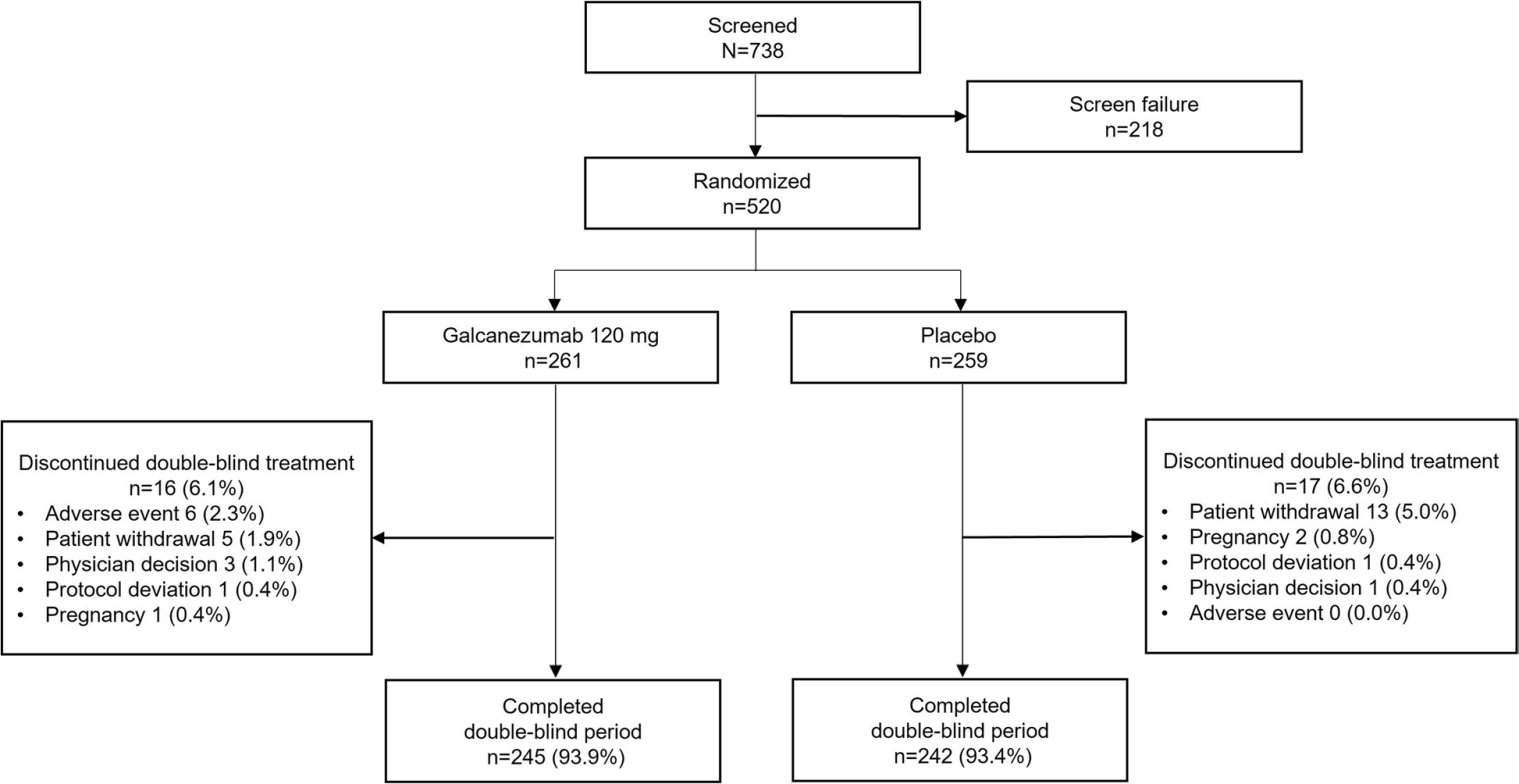
Patient disposition

A total of 738 patients entered study screening and 520 were randomized and received at least one dose of galcanezumab 120 mg (*N =* 261) or placebo (*N =* 259) (Fig. 2). The first patient was enrolled in July 2019, and the last patient completed the double-blind treatment period in July 2021. The completion rate for the double-blind treatment period was high and similar between the galcanezumab and placebo groups (93.9% and 93.4%, respectively). The most frequent reasons for discontinuing during the double-blind treatment phase were adverse events in patients assigned to galcanezumab (2.3%) and patient withdrawal in the placebo group (5.0%).

Demographics and disease characteristics were well balanced between the two groups at baseline (Table 1). Patients were predominantly female (73.8%) and Chinese (76.2%), with a mean (standard deviation [SD]) age of 37.0 (9.6) years. The mean time from migraine diagnosis to study enrollment was 12.6 years and 55.4% of patients had a baseline migraine frequency of ≥ 8 MHDs per month. At baseline, among all patients the mean number of MHDs was 8.2 days, the mean MSQ-Role Function-Restrictive score was 56.6, indicating moderately impaired functioning, the mean total MIDAS score was 46.7, indicating very severe disability, and the mean PGI-S score was 4.4, indicating moderate migraine severity. Less than half of patients (44.6%) had used prior migraine medications; the most common prior medications (> 5% of patients) were flunarizine (18.8%), topiramate (7.1%), herbal preparations (6.5%), amitriptyline (6.7%), and propranolol (5.4%).

**Table 1:** Demographics and baseline disease characteristics

	**Galcanezumab 120 mg (*N =* 261)**	**Placebo (*N =* 259)**
Age, years	37.2 (9.3)	36.8 (9.8)
Females, n (%)	188 (72.0)	196 (75.7)
Race, n (%)		
Asian	239 (91.6)	239 (92.3)
White	22 (8.4)	20 (7.7)
Body mass index, kg/m^2^	23.4 (3.8)	22.5 (3.2)
Region, n (%)		
China	198 (75.9)	198 (76.4)
India	41 (15.7)	41 (15.8)
Russia	22 (8.4)	20 (7.7)
MHDs per month, n (%)	8.2 (2.8)	8.3 (2.7)
MHDs with acute medication use per month	5.4 (4.9)	4.9 (4.5)
Migraine frequency, n (%)		
< 8	119 (45.6)	113 (43.6)
≥ 8	142 (54.4)	146 (56.4)
Duration of migraine illness, years	12.8 (9.2)	12.4 (8.2)
ICHD MHDs per month, n (%)	6.3 (3.3)	6.3 (3.2)
Headache days per month, n (%)	9.1 (3.3)	9.1 (2.9)
Migraine attacks per month, n (%)	5.5 (1.9)	5.6 (1.7)
MSQ score		
Total	61.7 (15.8)	62.1 (15.7)
Role Function-Restrictive	56.0 (15.2)	57.2 (15.2)
Role Function-Preventive	66.2 (18.5)	65.9 (17.8)
Emotional Function	69.0 (22.2)	68.5 (21.9)
PGI-S	4.4 (1.3)	4.3 (1.3)
MIDAS total score	47.7 (37.2)	45.6 (38.6)
≥ 1 prior migraine medication, n (%)	112 (42.9)	120 (46.3)

Data are presented as mean (standard deviation) unless otherwise specified

*ICHD* International Classification of Headache Disorders, *MHD* Migraine headache day, *MIDAS* Migraine Disability Assessment, *MSQ* Migraine Specific Quality of Life Questionnaire, *N* Number of patients in the analysis population, *n* number of patients within each specific category, *PGI-S* Patient Global Impression of Severity

### Efficacy

**Fig. 3:**
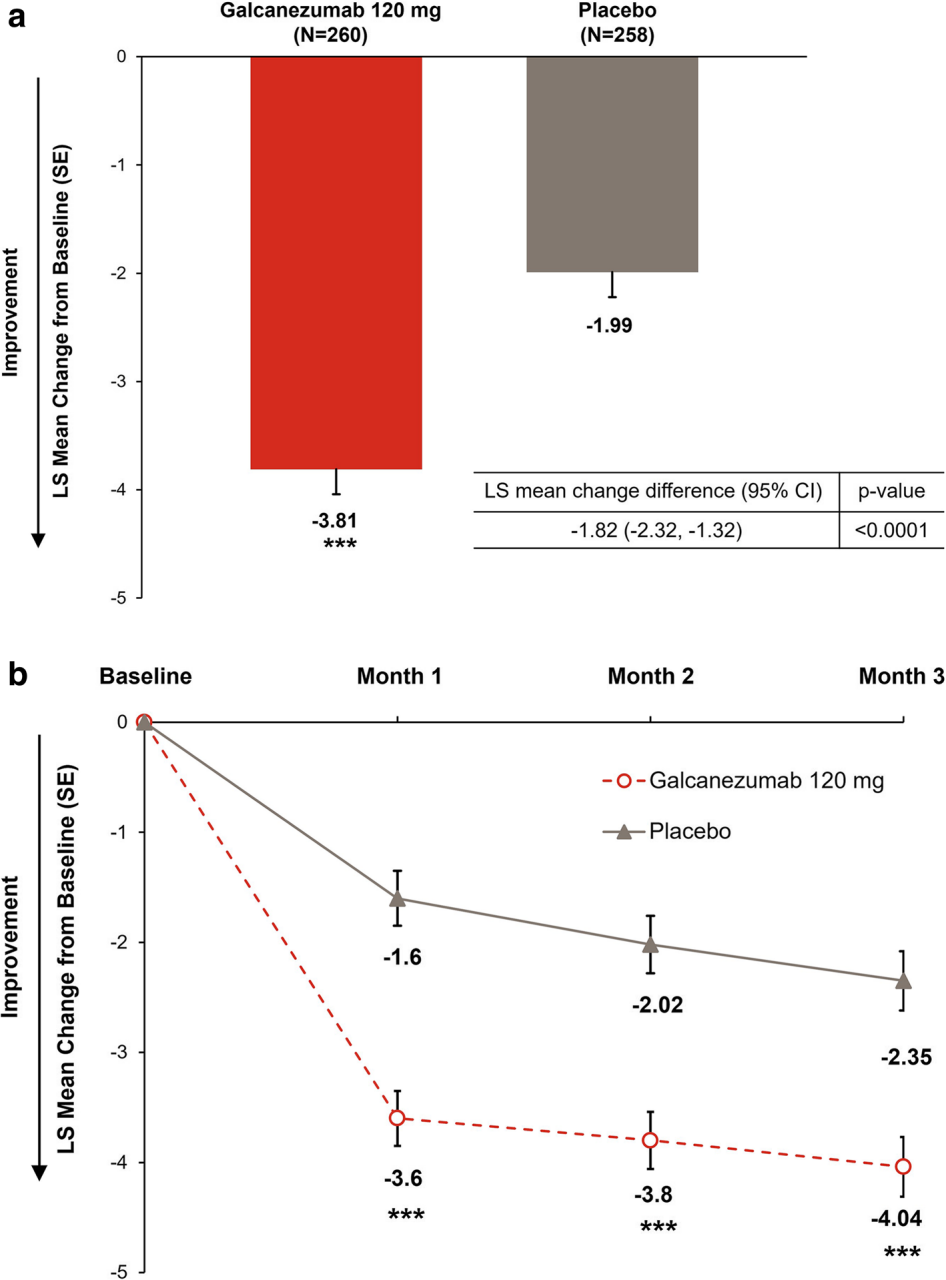
**a** Overall LS mean change from month 1 to 3 in monthly migraine headache days; **b** LS mean change at month 1 to 3 in monthly migraine headache days LS, least squares; SE, standard error. ***p < 0.0001 versus placebo

The primary endpoint of the study was met; galcanezumab resulted in a significantly (*p <* 0.0001) greater least squares (LS) mean reduction from baseline in monthly MHDs over 3 months compared with placebo (-3.81 days vs. -1.99 days; LS mean change difference: -1.82, 95% CI -2.32, -1.32, effect size = 0.63; Table 2; Fig. 3a). Galcanezumab demonstrated a rapid onset of effect with a significant improvement in the reduction in monthly MHDs versus placebo beginning at month 1 and continuing through to month 3 (*p <* 0.0001 at each month; Fig. 3b). The likelihood of having fewer weekly MHDs with galcanezumab compared with placebo was significant at week 1 (odds ratio 3.17; 95% CI 2.17, 4.62; *p <* 0.0001) and maintained at weeks 2, 3, and 4.

**Table 2:** Primary and secondary endpoints

Endpoint	Time frame	LS Mean Change (SE)		LS Mean Change Difference / Odds Ratio (95% CI)	*p*-value
		**Galcanezumab 120 mg**	**Placebo**		
Monthly MHDs	Month 1–3	-3.81 (0.23)	-1.99 (0.23)	-1.82 (-2.32, -1.32)^a^	< 0.0001
≥ 50% response rate	Month 1–3	54.9 (2.4)^b^	32.9 (2.3)^b^	2.48 (1.87, 3.29)^c^	< 0.0001
MSQ-RFR	Month 1–3	21.01 (0.85)	13.94 (0.88)	7.07 (5.20, 8.95)^a^	< 0.0001
≥ 75% response rate	Month 1–3	29.2 (2.1)^b^	12.7 (1.6)^b^	2.82 (2.01, 3.97)^c^	< 0.0001
100% response rate	Month 1–3	11.9 (1.4)^b^	3.9 (0.9)^b^	3.31 (1.99, 5.50)^c^	< 0.0001
Monthly MHDs treated with acute medication	Month 1–3	-2.49 (0.22)	-0.71 (0.22)	-1.78 (-2.25, -1.31)^a^	< 0.0001
MSQ-Total Score	Month 1–3	19.73 (0.81)	13.56 (0.84)	6.17 (4.39, 7.95)^a^	< 0.0001
MSQ-RFP	Month 1–3	18.79 (0.87)	12.76 (0.90)	6.03 (4.10, 7.95)^a^	< 0.0001
MSQ-EF	Month 1–3	17.88 (0.98)	13.72 (1.02)	4.16 (2.00, 6.32)^a^	0.0002
PGI-S	Month 3	-0.83 (0.09)	-0.61 (0.10)	-0.22 (-0.43, -0.02)^a^	0.0284
MIDAS Total Score	Month 3	-22.61 (2.96)	-10.18 (3.06)	-12.43 (-18.81, -6.05)^a^	0.0001

^a^LS mean change difference

^b^Model estimated rate

^c^Odds ratio

*CI* Confidence interval, *LS* Least squares, *MHD* Migraine headache day, *MIDAS* Migraine Disability Assessment, *MSQ-EF* Migraine Specific Quality of Life Questionnaire Emotional Function, *MSQ-RFP* Migraine Specific Quality of Life Questionnaire Role Function-Preventive, *MSQ-RFR* Migraine Specific Quality of Life Questionnaire Role Function-Restrictive, *PGI-S* Patient Global Impression of Severity, *SE* Standard error

**Fig. 4:**
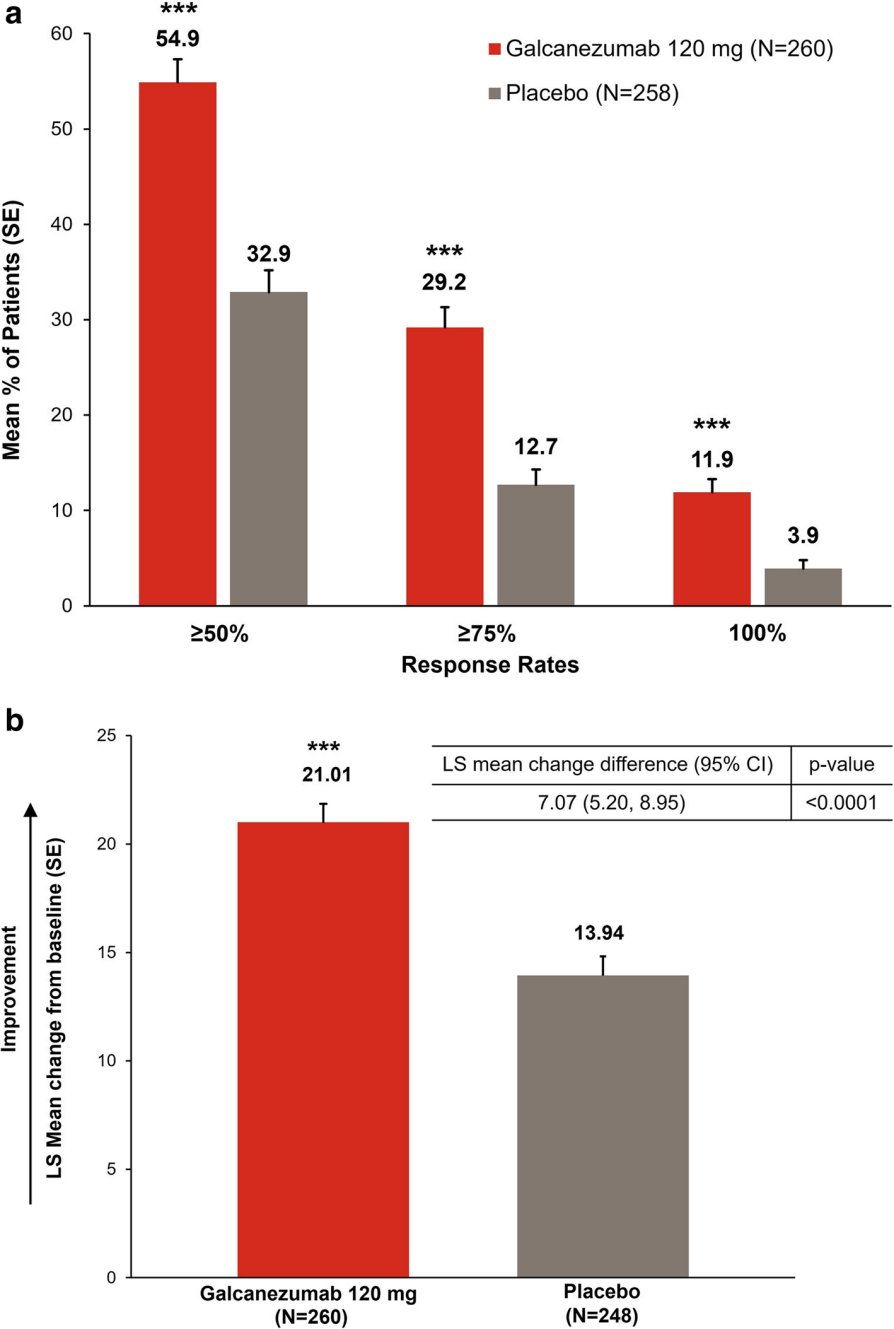
Key secondary endpoints: **a** mean percentage of patients with ≥ 50%, ≥ 75%, and 100% reductions in monthly migraine headache days; **b** LS mean change in the Migraine-Specific Quality of Life Questionnaire Role Function-Restrictive CI, confidence interval; LS, least squares; SE, standard error. ***p < 0.0001 versus placebo

All secondary endpoints were met. A significantly greater mean proportion of patients in the galcanezumab versus placebo group achieved ≥ 50%, ≥ 75% and 100% reductions from baseline in monthly MHDs over the 3-month study period (all *p <* 0.0001; Table 2; Fig. 4a). Additionally, the proportion of patients who maintained ≥ 50% response for all 3 months was significantly greater in the galcanezumab group compared with placebo group (29.6% vs. 12.4%; odds ratio 3.04; 95% CI 1.92, 4.81; *p <* 0.0001). Compared with placebo, galcanezumab significantly reduced the number of monthly MHDs requiring treatment with acute headache medication (*p <* 0.0001; Table 2).

The LS mean change of the MSQ Role Function-Restrictive score over 3 months was significantly greater with galcanezumab compared with placebo (*p <* 0.0001; Table 2; Fig. 4b). Galcanezumab was also associated with significantly greater LS mean changes of the MSQ total, Role Function-Preventive, and Emotional Function scores over 3 months compared with placebo (all *p <* 0.001; Table 2), indicating improvement of functional impairment due to migraine. There was a significantly greater mean reduction (improvement) from baseline in PGI-S rating at month 3 with galcanezumab compared with placebo (*p* = 0.0284; Table 2), indicating a reduction in severity of migraine condition. Galcanezumab significantly reduced the MIDAS total score from baseline to month 3 compared with placebo (*p* = 0.0001; Table 2), indicating a reduction in migraine disability.

### Safety

TEAEs were reported by 130 (49.8%) patients in the galcanezumab group and 112 (43.2%) patients in the placebo group (Table 3). The types and incidences of TEAEs were similar between galcanezumab and placebo. Among the patients with TEAEs, most (98.8%) reported these as mild or moderate in severity. The most frequently reported TEAE was injection site pain in both groups (galcanezumab 7.3% vs. placebo 6.2%). More patients in the galcanezumab versus placebo group reported injection site pruritis (5.0% vs. 0.0%), injection site reactions (3.8% vs. 0.4%), and injection site discomfort (2.3% vs. 0.0%). However, all TEAEs related to injection sites in the galcanezumab group were mild in severity, except for one patient who reported a moderate injection site reaction.

**Table 3:** Treatment-emergent adverse events occurring in ≥ 1.5% patients in the galcanezumab group

Preferred term, n (%)	Galcanezumab 120 mg (*N =* 261)	Placebo (*N =* 259)
Patients with ≥ 1 TEAE	130 (49.8)	112 (43.2)
Injection site pain	19 (7.3)	16 (6.2)
Upper respiratory tract infection	14 (5.4)	13 (5.0)
Injection site pruritis	13 (5.0)	0
Injection site reaction	10 (3.8)	1 (0.4)
Dizziness	9 (3.4)	6 (2.3)
Nasopharyngitis	7 (2.7)	9 (3.5)
Injection site discomfort	6 (2.3)	0
Protein urine present	6 (2.3)	4 (1.5)
Pyrexia	6 (2.3)	3 (1.2)
Abdominal discomfort	5 (1.9)	2 (0.8)
Aspartate aminotransferase increased	5 (1.9)	0
Injection site erythema	5 (1.9)	0
Alanine aminotransferase increased	4 (1.5)	0
Blood creatinine phosphokinase increased	4 (1.5)	0
Diarrhea	4 (1.5)	6 (2.3)
Pruritis	4 (1.5)	2 (0.8)

*TEAE* Treatment-emergent adverse event, *N* Number of patients in the analysis population, *n* number of patients within each specific category

SAEs were reported by two patients in the galcanezumab group (gastroenteritis and infected dermal cyst) and four patients in the placebo group (abortion threatened, COVID-19 pneumonia, hemorrhoids, and nasal septum deviation). However, none of the SAEs led to discontinuation of study treatment or were considered treatment-related by the investigator. No patients had died at the time of the data cutoff. TEAEs leading to treatment discontinuation occurred in one patient receiving placebo (spinal osteoarthritis) and six patients receiving galcanezumab (arrhythmia supraventricular, facial paralysis, hepatic function abnormal, rash, sudden hearing loss, and thyroid mass), all six events resolved and none of these were SAEs. Mean changes in vital signs and weight during the study were small with no clinically significant differences between the two groups.

### Immunogenicity

Among 507 evaluable patients, ADAs were present at baseline in 41 (15.8%) patients in the galcanezumab group and 33 (13.3%) patients in the placebo group. Treatment-emergent ADA positivity during the double-blind period was detected in 24 (9.3%) patients receiving galcanezumab and 3 (1.2%) of those receiving placebo. Of the 27 patients with treatment-emergent ADA positivity, neutralizing ADAs were present in 7 (2.7%) patients in the galcanezumab group and no patients in the placebo group. ADA titers were generally low; maximum treatment-emergent ADA titers ranged from 1:20 to 1:1280. There was no discernible effect of ADAs on treatment efficacy or tolerability.

## Discussion

Findings from the 3-month, double-blind period of the PERSIST study showed that galcanezumab 120 mg was significantly more effective than placebo across multiple endpoints, including the primary and all key secondary endpoints, in the prevention of episodic migraine in patients from China, India, and Russia. Galcanezumab significantly reduced the overall mean number of monthly MHDs compared with placebo over the 3-month treatment period, with rapid onset of action demonstrated by a significant effect at month 1. More than half of patients in the galcanezumab group had a ≥ 50% reduction in monthly MHDs, an established benchmark for a clinically meaningful result [25]. The mean number of monthly MHDs with use of headache medication was also significantly reduced with galcanezumab versus placebo, which has clinical importance in terms of preventing the development of medication overuse headache [26]. Furthermore, galcanezumab improved functioning and reduced the disability burden compared with placebo, as demonstrated by improvements in MSQ, PGI-S, and MIDAS scores.

The global average prevalence rate of migraine is approximately 11%, and there are huge unmet needs for the preventive treatment of migraine [27]. The PERSIST study is the first clinical trial designed to investigate galcanezumab primarily in an Asian population. The results of the present study are consistent with findings from two previous phase 3 studies of galcanezumab in patients with episodic migraine, which were conducted in the United States, Canada and Puerto Rico (EVOLVE-1) [17] and North America, Europe, Argentina, Israel, Korea, Taiwan, and Mexico (EVOLVE-2) [18]. The difference in LS mean change in the number of monthly MHDs with galcanezumab 120 mg versus placebo in the present study (-1.82) was similar to that observed in the EVOLVE-1 (-1.9) [17] and EVOLVE-2 (-2.02) [18] studies. Differences between the present study and the previous two studies should however be noted, including a shorter duration of treatment (3 months vs. 6 months, respectively), and a patient population with lower mean age (37.0 years vs. 40.7 and 41.9 years, respectively), fewer mean monthly MHDs (8.2 days vs. 9.2 and 9.1 days, respectively), and a shorter duration of migraine illness (12.6 years vs. 20.2 and 20.0 years, respectively) at baseline.

Galcanezumab showed acceptable tolerability in the present study and was associated with low rates of SAEs and discontinuations due to TEAEs, in accordance with the known safety profile based on previous phase 3 studies [17–20]. Injection site pain was the most common TEAE in both the galcanezumab and placebo groups. The incidence of injection site pruritis, injection site reaction, and injection site discomfort was numerically increased with galcanezumab versus placebo. However, these TEAEs were all mild or moderate in severity. Furthermore, there was no evidence that hypersensitivity events or TEAEs related to injection sites were mediated by treatment-emergent ADAs.

In addition to the data from the EVOLVE-1, EVOLVE-2 and PERSIST studies in patients with episodic migraine, the REGAIN study showed that galcanezumab was efficacious, safe, and well tolerated for the preventive treatment of chronic migraine, with a LSM change difference of -2.1 MHDs between galcanezumab 120 mg and placebo [19]. Furthermore, the CONQUER study showed that galcanezumab was superior to placebo in the preventive treatment of migraine and was safe and well tolerated in patients for whom two to four drug categories of preventive treatments had failed, with a LSM change difference of -3.1 MHDs compared with placebo [20].

In addition to the data from the present randomized controlled study, that include primarily Asian patients, regional data on galcanezumab are emerging from real-world studies [28, 29]. Results were recently reported from a Korean prospective registry study, which included 22 patients who received galcanezumab for the prevention of episodic migraine [28]. Among these 22 patients, a ≥ 50% reduction in the number of moderate/severe headache days at 3 months was observed in 54.5% of patients, and a ≥ 30% and ≥ 75% reduction was seen in 59.1% and 27.3% of patients, respectively. In an Italian multicenter cohort study (GARLIT), 33 patients received galcanezumab for the prevention of high-frequency episodic migraine [29]. The proportions of patients with ≥ 50% and ≥ 75% reductions in monthly migraine days at 3 months were 67.6% and 35.3%, respectively. Therefore, the response rates observed in the PERSIST study are comparable to those reported in these real-world studies.

Limitations of this study included the lack of testing to determine whether galcanezumab could be effective as an adjunctive treatment combined with other preventive medications. Furthermore, the exclusion of patients with serious medical or psychiatric conditions, high body mass index, substantial opioid use, and high risk for major cardiovascular events may limit the generalizability of the results. The duration of double-blind treatment was also shorter than in the previous studies (3 months vs. 6 months, respectively), [17, 18] and may not have been sufficient to detect long-term risks. However, the study is on-going, and analysis of the open-label and post-treatment periods will provide insight into the benefits and risks of longer-term use of galcanezumab in this patient population.

## Conclusion

Galcanezumab 120 mg given once monthly was effective and well tolerated in patients with episodic migraine from China, India, and Russia. These findings were consistent with previous data on galcanezumab in patients with episodic migraine from previous pivotal phase 3 studies [17, 18].

## Data Availability

Lilly provides access to all individual participant data collected during the trial, after anonymization, except for pharmacokinetic or genetic data. Data are available upon reasonable request. Access is provided after a proposal has been approved by an independent review committee identified for this purpose and after receipt of a signed data sharing agreement. Data and documents, including the study protocol, statistical analysis plan, clinical study report, blank or annotated case report forms, will be provided in a secure data sharing environment. For details on submitting a request, see the instructions provided at www.vivli.org.

## Abbreviations

ADA: Antidrug antibody
CGRP: Calcitonin gene-related peptide
ePRO: Electronic patient-reported outcomes
ICHD: International Classification of Headache Disorders
LS: Least squares
MHDs: Migraine headache days
MIDAS: Migraine Disability Assessment
MMRM: Mixed model repeated measures
MSQ: Migraine-Specific Quality of Life Questionnaire
PGI-S: Patient Global Impression of Severity
SAE: Serious adverse event
SD: Standard deviation
TEAE: Treatment-emergent adverse event
